# Torque regulation is affected by joint angle during isometric contraction in young male adults

**DOI:** 10.1038/s41598-026-42754-y

**Published:** 2026-03-10

**Authors:** J. H. Oliveira, J. S. Gomes, P. Bauer, P. Pezarat-Correia, J. R. Vaz

**Affiliations:** 1 Neuromuscular Research Laboratory, Faculty of Human Kinetics, University of Lisbon, Lisbon, Portugal; 2 CIPER, Faculty of Human Kinetics, University of Lisbon, Lisbon, Portugal; 3 Egas Moniz Center for Interdisciplinary Research (CiiEM), Egas Moniz School of Health & Science, 2829-511 Almada, Portugal

**Keywords:** Complexity, Variability, Entropy, Force control, Muscle length, Electromyography - EMG, Physiology

## Abstract

Research demonstrates that the magnitude of torque variability is influenced by joint angle. However, complexity-based measures seem to provide distinct information than the traditional magnitude-based measures of variability, being more sensitive to detect subtle changes in physiological outputs’ dynamics. Therefore, this study aimed to investigate the effect of joint angle on torque complexity and intermuscular coordination. Twenty-five male adults performed a sustained isometric knee extensor task at 110% of their end-test torque for 30 s at five different joint angles. Torque complexity and variability were assessed using Sample Entropy (SampEn) and Coefficient of Variation (CV), respectively, while intermuscular coordination was evaluated through Co-Contraction Index (CCi). Results revealed that SampEn at OA−30º was significantly lower than at OA (*p* = 0.014) and OA + 15º (*p* = 0.022), indicating reduced torque complexity at shortened muscle lengths. CV and CCi exhibited similar behaviors by presenting a trend for a U-shape relationship with joint angle. Our findings suggest a decrease in the individuals’ capacity to regulate the torque production with the shortening of the knee extensors which can possibly be explained by the mechanical properties of the muscle and central and peripheral neuromuscular factors or, most importantly, by the complex interaction between all the mechanisms involved in torque regulation.

## Introduction

Healthy physiological systems are characterised by the interaction of multiple components and feedback loops that operate over a range of temporal and spatial scales^1^ resulting in outputs presenting constant inherent fluctuations^2^.

In the neuromuscular system, such fluctuations in torque output are relevant as they influence the individual’s capacity to achieve a desired force or produce an intended movement trajectory^3^. Specifically, the temporal structure of torque fluctuations, i.e. torque complexity, has been proposed as a fundamental indicator of the neuromuscular system’s ability to explore and adapt control strategies to meet the demands of a given motor task, i.e. motor control^4–7^. For instance, Ravi and colleagues^8^ found that individuals recovered more rapidly from a locomotor perturbation when synchronizing to a metronome with optimal complexity incorporated in its temporal structure, compared to a rigid stimulus lacking such complexity, highlighting the role of complexity in enhancing individuals’ adaptability and functional capacity^9,10^.

Moreover, the literature has demonstrated that ageing, injury and disease states lead to a loss of complexity in the time series of several physiological outputs such as heart rate^2^, gait^11^, postural control^12^ and torque^13^, resulting in less adaptable systems, i.e., systems with diminished capacity to flexibly adapt to environmental perturbations. More recently, neuromuscular fatigue has also been shown to induce a loss of torque complexity, with consequences at the motor control level, compromising the neuromuscular system’s ability to rapidly and accurately adjust motor output in response to changes in task demands^9,14–18^. Importantly, this loss of torque complexity is influenced by the contraction nature^19^ and intensity^20^, particularly above the critical torque^10^.

Although torque complexity is known to be influenced by several biological and physiological processes, less is known about its susceptibility to the impact of muscle mechanics. It is well accepted that muscle mechanics influence the capacity of muscle to produce force. Specifically, the literature demonstrates that the ability of the neuromuscular system to produce maximal force is influenced by the joint angle in an inverted U-shape relationship, reaching the highest levels at the muscle’s optimal angle (OA)^21–23^.

Interestingly, Sosnoff and colleagues^23^ have raised the hypothesis that the torque control could also be influenced by joint angle. The authors have shown that the relative variability decreases as the degree of knee flexion increases, with the greatest variability at 15º of knee flexion, i.e. with the knee extensors in a shortened position. However, in this study, the authors used the traditional magnitude-based measures of variability such as standard deviation and coefficient of variation to assess torque control. However, previous research suggested that these measures provide distinct information than the complexity-based measures, which have been suggested to have the capacity to detect differences in the dynamics of outputs that magnitude-based measures are insensitive to^24–27^.

Although an effect of joint angle on the magnitude of variability may be acknowledged, its specific influence on torque complexity remains to be elucidated. Understanding this potential influence may provide deeper insights into the neuromechanical basis of torque complexity. Therefore, the present study aimed to investigate the effect of knee joint angle on the complexity of torque output produced by knee extensors. We hypothesised an inverted U-shape relationship between torque complexity and knee joint angle, with the highest values being reached at the optimal angle. Conversely, considering that, using degrees of flexion of 15–90º, previous research suggested a decrease in force variability as a function of joint angle, we hypothesised that, for higher degrees of knee flexion, the torque variability would be greater and, hence, a U-shape relationship between coefficient of variation and joint angle would be observed.

Additionally, although the literature suggests an integrated response of both peripheral and central processes, the neurophysiological mechanisms underpinning the loss of torque complexity are not fully understood^17,18^. Indeed, one fundamental question in motor control is to understand how the nervous system interacts with muscles, through intra and intermuscular coordination mechanisms, to generate appropriate joint torques and to control the movement. Specifically, co-contraction – the simultaneous activation of agonist and antagonist muscle groups^28^– is a mechanism of intermuscular coordination used by neuromuscular system to increase the joint stiffness and stability to achieve movement accuracy, or to minimize the impact of perturbations during tasks in unpredictable or unstable environment^29–34^. Therefore, co-contraction has long been studied in human motor control, with the literature suggesting that it could be potentially influenced by joint angle^35,36^. Thus, although this was a secondary aim of the study, we also aimed to investigate the joint angle effect on co-contraction index. We hypothesised a U-shape relationship between the co-contraction index and joint angle, with co-contraction reaching the lowest values at optimal angle and highest values at both shortened and lengthened positions.

## Materials and methods

### Participants

Twenty-five young and healthy male adults (age:22.8 ± 3.3; height:1.77 ± 0.06 m; body mass:70.5 ± 7.0 kg; body mass index:22.5 ± 1.6 kg/m^2^) without any neurological or motor disorders were included in the present study. All participants provided written informed consent, previously approved by the Ethics Committee of the Faculty of Human Kinetics (approval number #3/2022) and in accordance with the Declaration of Helsinki. Participants were instructed to avoid moderate-to-vigorous physical activity and resistance training for the completion study’s duration.

### Experimental design and protocol

The participants completed two laboratory sessions, separated by at least 48 h, at the same time of the day to minimise circadian effects on muscle force^37^. Both sessions started with a warm-up that included submaximal isometric and isokinetic knee extensions.

During the first session, participants performed a series of assessments that encompassed the determination of their optimal angle (OA) for the knee extension movement, the quantification of their Peak Torque (PT) during Maximal Voluntary Isometric Contraction (MVIC) and the evaluation of their end-test torque (ETT).

To determine de OA, participants were asked to complete five consecutive, maximal voluntary isokinetic concentric contractions at 60º/s through an 110º range of movement. OA was defined as the joint position in which the highest torque value (i.e., peak torque) was achieved during the test.

After a 10-minute rest, participants performed three MVIC lasting 3–5 s with 60 s of rest between trials to establish their PT. During the test, participants were instructed to exert their maximum force as fast as possible with strong verbal encouragement.

Following PT assessment, ETT was evaluated using a 5-minutes all-out test comprising 60 intermittent MVIC (3s contraction, 2s rest)^38^. ETT was defined as the mean of the PT obtained during the last six contractions. Participants were verbally instructed to “push” and to “stop” and strongly encouraged to maximize torque production during each contraction despite the expected decline in the capacity to produce torque, but were not informed about the elapsed time or the number of contractions remaining, and no visual feedback was provided. ETT was then normalised to each participant’s PT to define the submaximal intensity for the second session. Finally, before completing the first session, participants performed a series of sustained submaximal isotonic contraction to get familiarised with the testing protocol.

**Fig. 1:**
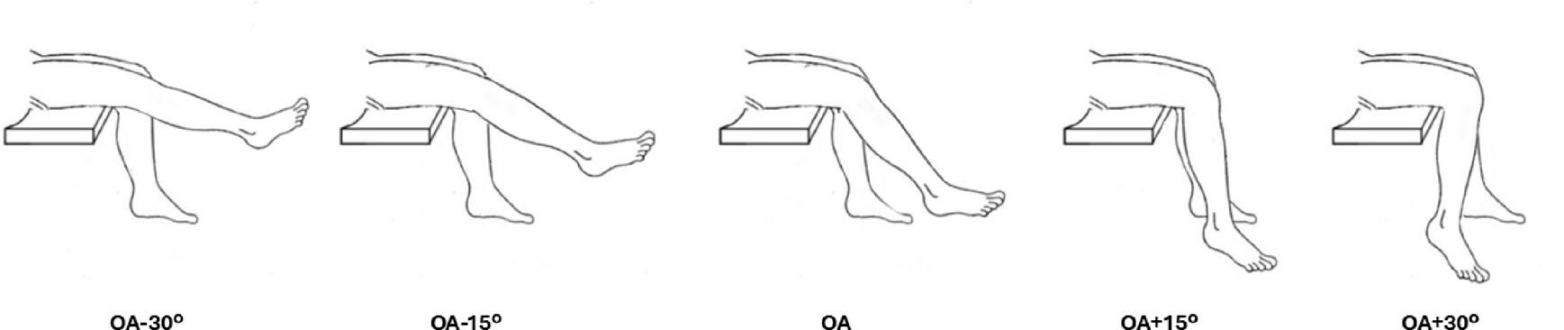
Schematic figure of joint positions (OA + 30º, OA + 15º, OA, OA-15º, OA-30º). OA stands for optimal angle.

The second session involved experimental testing at five joint angles: OA, OA−30º, OA−15º, OA + 15º and OA + 30º these angles were individualised for each participant based on the OA determined in session one, allowing for systematic testing across a range of muscle lengths from shortened (OA−30º) to a lengthened (OA + 30º) positions Fig. 1.

At each testing angle, participants first completed a series of brief MVIC to determine PT at that specific position. Subsequently, they performed two trials, with 1-minute rest, of a 30-second submaximal hold isometric task, consisting in sustain a submaximal intensity calculated by multiplying the PT obtained at each testing angle by the individual relative ETT plus 10%, ensuring the task was performed above the critical torque as recommended in the literature^10^. The isotonic mode of the isokinetic dynamometer was used, and lever arm was placed at the desired articular position by the researcher. Then, participants were instructed to maintain the articular position and to match their instantaneous torque with a target line superimposed on a display in front of them, equivalent to the resistance applied by the isokinetic dynamometer. Trials were repeated if the joint angle varied by more than 10º from the prescribed angle. The order of testing angles was randomized.

### Data collection

#### Dynamometry

An isokinetic dynamometer (Biodex Medical System Pro 3, Shirley, NY, USA), initialised and calibrated according to the manufacturer’s instructions, was used for all testing procedures. Participants were seated with hip and knee angles set at 90º (with the full extension being 0°), and the lateral femoral epicondyle of the dominant leg aligned with the lever arm’s axis of rotation. The lower leg was attached to the lever arm above the malleoli with a velcro strap. Additional straps were firmly secured across waist and shoulders to minimise extraneous movement and prevent compensations from hip extensors during the contractions. Torque data were sampled at 1000 Hz through Biopac MP100 (Biopac Systems Inc., California, USA) interfaced with a personal computer, recorded in Acknowledge (Version 3.9.1. Biopac Systems, Inc., California, USA) and further exported to Matlab^®^ R2023a (The MathWorks Inc., Natick, MA, USA).

#### Electromyography

During the second session, surface electromyography (EMG) signals were recorded using five bipolar electrodes (Trigno Avanti, Delsys, Natick, USA) placed on *Vastus Lateralis* (VL), *Vastus Medialis* (VM), *Rectus Femoris* (RF), *Biceps Femoris* (BF) and *Semitendinous* (ST). Electrode placement followed the Surface EMG for Non-Invasive Assessment of Muscles (SENIAM) recommendations^39^, with sensors aligned to muscle fibers and fixed with specially designed detection surfaces. Additionally, tape was used to firmly secure the electrodes to the participants’ skin to prevent movement artifacts. To improve signal conduction, skin was shaved and cleaned with alcohol prior to the electrodes’ placement. EMG signals were preamplified and band-pass filtered between 20 and 450 Hz, while sampled at 1000 Hz, and collected through EMG Works software (Delsys, Natick, USA).

### Data analysis

All data from the experimental protocol were processed using code written in Matlab^®^ R2023a (The MathWorks, Natick, MA, USA).

Torque signals were first low-pass filtered (Butterworth 10 Hz, 4th order). For MVIC trials, PT was determined as the highest torque value obtained during each contraction. For statistical purposes, the highest PT values amongst the trials were used for statistical analysis. Regarding submaximal trials, the signals were downsampled to 100 Hz following a power spectral analysis that revealed a maximal frequency across participants of 12.48 Hz. This downsampling complied with the recommendations of a sampling frequency five times greater than the highest frequency of interest in the time series^40^. Further, to analyse only the time the participant matched the targeted torque, the signals were cropped to remove the ascending and descending components of the hold isometric contractions. Then, from the cropped signals, the magnitude and temporal structure of variability were calculated. The magnitude of variability was assessed through the Coefficient of Variation (CV), calculated normalizing the standard deviation by the mean. The Sample Entropy (SampEn) was used to determine the temporal structure of the torque output^41^. SampEn is a measure of the signal’s regularity, that is, a measure that determines the probability that short sequences of data points are repeated throughout a temporal sequence of points. For instance, a regular signal, i.e. a signal characterised by perfectly repeatable time series present a SampEn value equal to zero, while a random signal has a SampEn value converging towards infinity^41^. In this study, a pattern length (m) of 2, error tolerance (r) of 0.2 and data length (N) of 3000 data points (i.e., 100 Hz x 30 s) were selected and used in the determination of SampEn values following recent recommendations^42,43^. These input values have shown optimal reliability when applied to all trials and participants^44^. SampEn and CV were extracted from the exact same previously cropped signal and the average of the two trials in each angle was used for statistical analysis.

Regarding EMG, raw EMG signals were band-pass filtered (20–490 Hz), rectified, smoothed with a low-pass filter (Butterworth 12 Hz, 4th order) and normalised to the average EMG amplitude during a 100ms window centered on the EMG peak during the MVIC in which PT was obtained. EMG signals were then cropped similarly to the torque data. Co-contraction indices (CCi) were calculated for six quadriceps-hamstring muscle pairs (VL-ST, VL-BF, VM-ST, VM-BF, RF-ST, RF-BF) using the method described by Rudolph et al.^45^. Average EMG amplitude was also determined for each muscle during the submaximal tasks.

### Statistical analysis

All statistical analyses were performed using Jamovi (Version 1.6. Sydney, Australia). Standard descriptive statistics including mean, standard deviation and 95% confidence interval were used to summarise the data. Normality for all variables was tested using Shapiro-Wilk tests. One-way repeated measures ANOVA were conducted to test the effect of joint angle on torque parameters (SampEn, CV, PT) and intermuscular coordination (CCi). Mauchly’s test was implemented to test sphericity, and the Greenhouse–Geisser correction was used when not verified. When normality was violated, a Friedman’s test was used. Tukey-adjusted 95% paired-samples confidence intervals (95% CI) were used to identify specific differences. Statistical significance was set at *p* < 0.05.

## Results

### Torque-related parameters

All data regarding peak torque and the magnitude (CV) and temporal structure (SampEn) of torque fluctuations are presented in Table 1.

**Table 1:** Joint angle effect on peak torque and magnitude and temporal structure of torque output fluctuations.

Condition	Angle (º)	Peak Torque (*N*.m)	SampEn	CV
OA-30º	39.3 ± 6.3	168.6 ± 47.9^a, b,c, d^ [149.8–187.4]	0.809 ± 0.171^b, c^ [0.741–0.876]	5.11 ± 1.20^a^ [4.64–5.58]
OA-15º	54.3 ± 6.3	246.1 ± 54.3^b, c^ [224.8–267.4]	0.875 ± 0.183 [0.803–0.946]	4.24 ± 0.88^d^ [3.90–4.59]
OA	69.3 ± 6.3	321.4 ± 65.2^c, d^ [295.8–346.9]	0.920 ± 0.200 [0.841–0.998]	4.62 ± 1.29 [4.11–5.13 ]
OA+15º	84.3 ± 6.3	298.5 ± 66.1^d^ [272.6–324.4]	0.916 ± 0.169 [0.849–0.982]	4.54 ± 1.00 [4.14–4.93]
OA+30º	99.3 ± 6.3	220.7 ± 48.1 [201.8–239.6]	0.913 ± 0.162 [0.849–0.976]	5.22 ± 2.00 [4.42–6.02]

Mean ± SD [95% Confidence Interval]. Symbols indicate a statistically significant difference compared to the following: ^a^OA-15º, ^b^OA, ^c^OA+15º, ^d^OA+30º.

For maximal task, we observed a significant effect of joint angle on PT (F_(2.02, 48.82)_ = 85.3, *p* < 0.001, ${\eta}_{p}^{2}$ = 0.780). Post hoc comparisons showed PT in OA-30º to be lower than in OA-15º, OA, OA+15º and OA + 30 (all *p* < 0.001), as well as PT in OA-15º to be lower than in OA (*p* < 0.001) and OA+15º (*p* = 0.001); conversely, the post hoc results demonstrated the PT in OA to be significant higher than OA+15º (*p* = 0.047) and OA + 30 (*p* < 0.001), and the PT in OA+15º to be higher than OA+30º (*p* < 0.001).

**Fig. 2:**
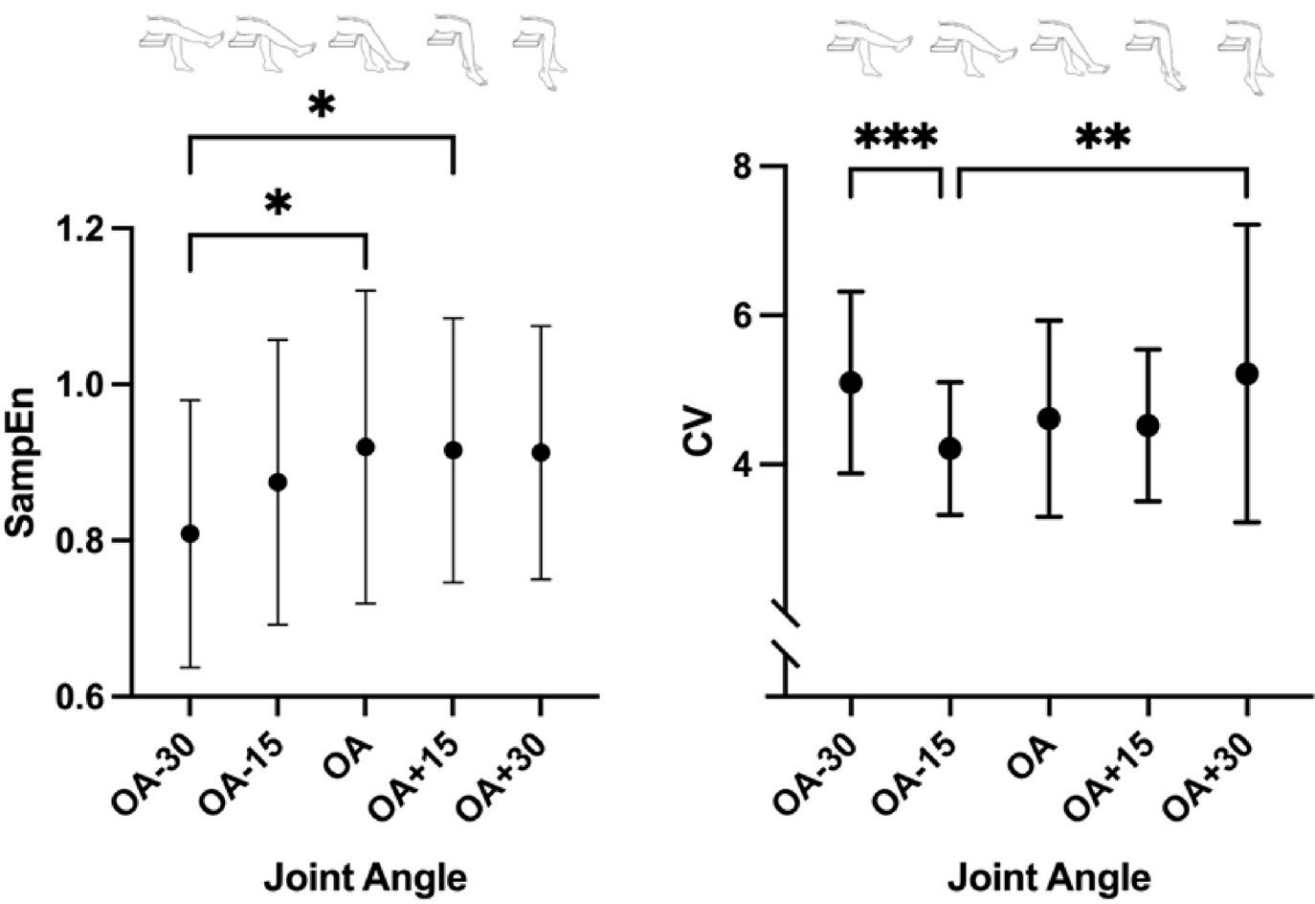
Effect of joint angle on temporal structure and magnitude of torque variability. Data are presented as mean and standard deviation for each joint angle. SampEn stands for Sample Entropy, and CV for Coefficient of Variation. *, ** and *** represent *p* < 0.05, *p* < 0.01 and *p* < 0.001, respectively.

For the temporal structure of torque output fluctuations, a significant effect of joint angle was observed on SampEn (F_(4, 96)_ = 5.22, *p* < 0.001, ${\eta}_{p}^{2}$ = 0.176). Post hoc comparison revealed SampEn in OA-30º to be significantly lower than OA (*p* = 0.014) and OA+15º (*p* = 0.022), as demonstrated in Fig. 2.

Regarding the magnitude, it was observed that CV was affected by joint angle (Χ^2^_(4)_ = 22.4, *p* < 0.001, W = 0.224). Post hoc comparisons showed CV in OA-30º to be higher than in OA-15º, and CV in OA-15º (*p* < 0.001) to be lower than in OA + 30º (*p* = 0.0018), as depicted in Fig. 2.

### EMG-related parameters

All EMG data are summarized in Table 2.

#### Co-contraction Index

**Fig. 3:**
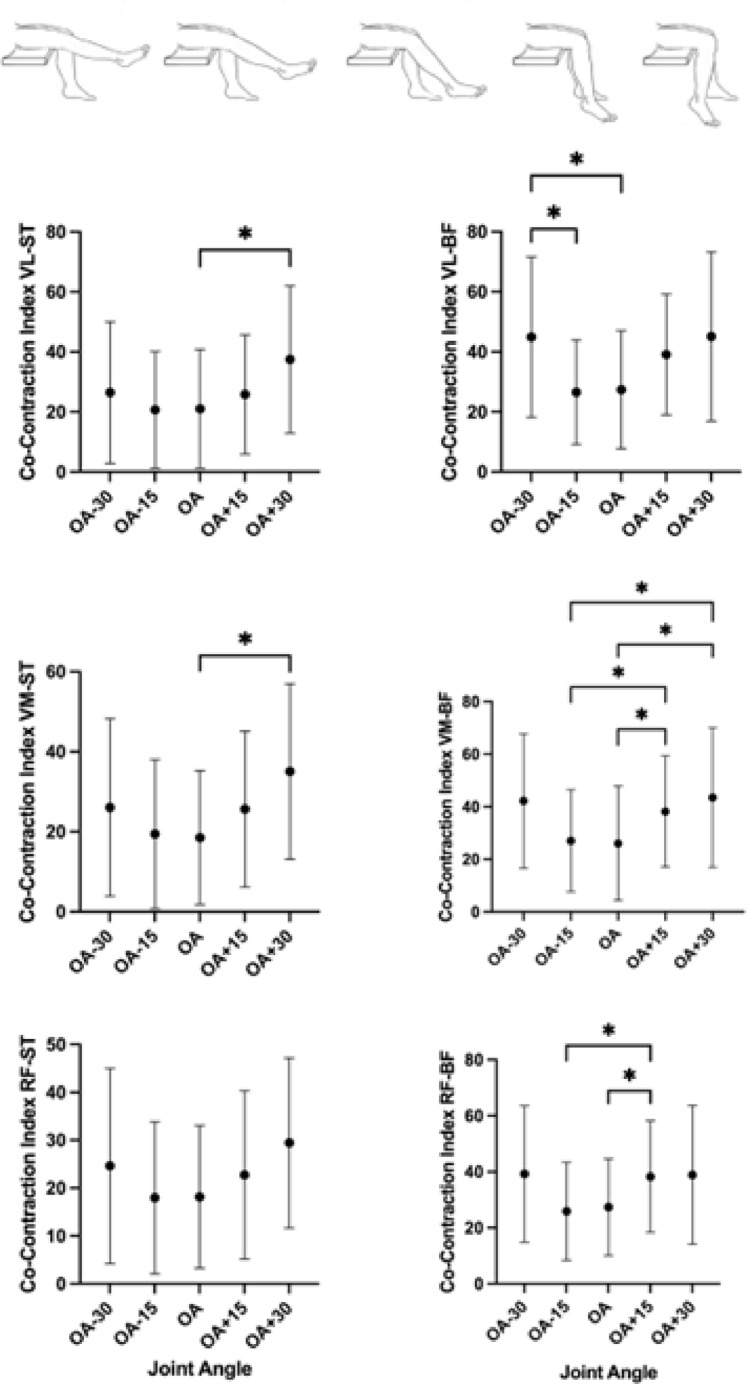
Joint angle effect on co-contraction Index. VL stands for *vastus lateralis*, VM for *vastus medialis*, RF for *rectus femoris*, ST for *semitendinosus* and BF for *biceps femoris*. Data are presented as mean and standard deviation for each joint angle. * represents *p* < 0.05.

CCi was significantly affected by the joint angle in the following pairs of muscles (Fig. 3): VL-ST (Χ^2^_(4)_ = 12.38, *p* = 0.015, W = 0.135), VL-BF (Χ^2^_(4)_ = 18.92, *p* < 0.001, W = 0.206), VM-ST (Χ^2^_(4)_ = 11.51, *p* = 0.021, W = 0.125), VM-BF (Χ^2^_(4)_ = 21.01, *p* < 0.001, W = 0.228) and RF-BF (Χ^2^_(4)_ = 19.03, *p* < 0.001, W = 0.206). Post hoc comparisons demonstrated the VL-ST and VM-ST in OA + 30 to be significantly higher than in OA (*p* = 0.015). For the pair VL-BF, the CCi in OA-30º was shown to higher than in OA-15º (*p* = 0.021) and OA (*p* = 0.038). Regarding the pair VM-BF, we observed the CCi in OA + 30º and OA + 15º to be higher than in OA (*p* = 0.021 and *p* = 0.028, respectively) and OA-15º (*p* = 0.021 and *p* = 0.028, respectively). For RF-BF pair, the results shown the Cci in OA + 15º to be higher than in OA and OA-15º (*p* = 0.021).

For the pair RF-ST, no significant effect of joint angle was observed in CCi (Χ^2^_(4)_ = 9.32, *p* = 0.054, W = 0.101).

**Table 2:** Surface EMG-related parameters across five different joint angles.

		OA-30º	OA-15º	OA	OA+15º	OA+30º
EMG amp	VL	41.3 ± 16.0 [34.8–47.7]	35.2 ± 13.4 [29.9–40.4]	33.3 ± 15.2 [27.3–39.2]	35.2 ± 13.2 [30.0–40.5]	36.8 ± 13.4 [31.6–42.1]
	VM	33.8 ± 16.7 [27.1–40.5]	34.5 ± 15.6 [28.4–40.6]	28.9 ± 14^c^ [23.3–34.4]	38.3 ± 17.3 [31.4–45.2]	36.8 ± 16.7 [30.2–43.3]
	RF	34.0 ± 19.1^b^ [26.4–41.6]	32.8 ± 17.8 [25.8–39.7]	27.0 ± 11.5^c^ [22.5–31.6]	34.4 ± 14.2 [28.7–40.0]	34.1 ± 18.4 [27.0–41.3]
	ST	18.2 ± 17.2 [11.3–25.1]	13.3 ± 11.2 [8.9–17.7]	14.7 ± 15.6^d^ [8.6–20.8]	18.2 ± 13.7 [12.8–23.7]	26.0 ± 19.7 [18.3–33.8]
	BF	32.1 ± 19.6^a^ [24.2–39.9]	18.2 ± 10.1^c, d^ [14.2–22.2]	20.7 ± 13.3 [15.5–25.9]	28.1 ± 14.6 [22.3–34.0]	32.8 ± 19.7 [25.1–40.5]
CCi	VL-ST	25.5 ± 23.6 [16.1–34.9]	20.0 ± 19.0 [12.6–27.4]	19.7 ± 19.5^d^ [12.0–27.3]	26.8 ± 19.9 [18.8–34.7]	36.9 ± 24.8 [27.2–46.6]
	VL-BF	47.0 ± 28.0^a, b^ [35.7–58.2]	26.4 ± 16.7 [19.9–33.0]	28.9 ± 21.2 [20.6–37.2]	39.3 ± 19.7 [31.4–47.2]	46.5 ± 28.1 [35.5–57.6]
	VM-ST	25.2 ± 22.2 [16.3–34.0]	19.5 ± 18.5 [12.2–26.7]	17.4 ± 16.5^d^ [11.0–23.9]	26.5 ± 19.5 [18.7–34.3]	34.4 ± 22.2 [25.7–43.1]
	VM-BF	44.3 ± 27.0 [33.5–55.1]	26.8 ± 18.6^c, d^ [19.5–34.1]	27.5 ± 22.6^c, d^ [18.7–36.4]	40.9 ± 24.6 [31.1–50.8]	46.1 ± 28.9 [34.8–57.4]
	RF-ST	23.8 ± 20.4 [15.6–31.9]	18.1 ± 16.0 [11.9–24.4]	17.1 ± 14.7 [11.3–22.8]	23.8 ± 18.0 [16.6–31.1]	29.4 ± 18.6 [22.1–36.8]
	RF-BF	38.9 ± 23.8 [29.4–48.5]	26.0 ± 16.7^c^ [19.4–32.5]	28.9 ± 19.0^c^ [21.4–36.3]	40.0 ± 21.2 [31.5–48.4]	40.5 ± 25.1 [30.6–50.3]

Mean ± SD [95% Confidence Interval]. Symbols indicate a statistically significant difference compared to the following: ^a^OA-15º, ^b^OA, ^c^OA+15º, ^d^OA+30.

## Discussion

The present study investigated how torque complexity is influenced by joint angle, specifically whether the temporal structure of torque output fluctuations (i.e. torque complexity) would be influenced by changes in muscle length through variations in joint angle. We hypothesised an inverted U-shape relationship with torque complexity values peaking at the optimal angle (OA) and decreasing when the knee extensors’ muscles were either shortened or lengthened. Our results partially support the hypothesis since torque complexity reached the highest values at OA and subsequently decreased in the shortened position but remain unchanged in the lengthened muscle position. Furthermore, as hypothesised, we observed an increased magnitude of variability, measured through CV, in both flexed and extended positions. Additionally, we aimed to investigate the effect of joint angle on intermuscular coordination parameters, hypothesising a U-shape relationship between co-contraction index (CCi) and joint angle. While increases in CCi were primarily evident with muscle lengthening, a global U-shape trend was observed with CCi presenting the lowest values at OA, and the highest values at either the shortened or lengthened positions.

As previously described by Bernstein^46^, the multiple degrees of freedom of the body, including joints, muscles, and the nervous system, combine with environment constraints such as external forces, and task constraints to produce countless patterns, forms, and strategies of movement. Therefore, the redundancy of the system allows for the use of multiple strategies to accomplish any given task, resulting in the inherent variability that characterises motor performance. In the present study, altering joint angle introduced a mechanical constraint and, importantly, we observed that placing the knee extensors in a shortened position leads to a decrease in torque complexity, suggesting a decline in the neuromuscular system’s capacity to adapt the motor output to changes in the task constraints, resulting in more predictable, less flexible motor output. Moreover, we observed the sample entropy to reaching its peak at the OA which aligns with prior findings demonstrating that force production is maximal at muscles’ OA^22,47–49^ due to the optimal actin-myosin overlap and calcium sensitivity observed at this angle, increasing the number of formed cross bridges^50^.

Applying this rationale to motor control tasks, we could hypothesise that the enhanced adaptability at OA may result from an increased number of available motor solutions, allowing the neuromuscular system to flexibly respond to the task demands. Conversely, when the knee joint is in a flexed position (OA-30º), the consequent reduced muscle length likely limits the actin-myosin overlaps, decreasing the available motor strategies and, thus, torque complexity.

Interestingly, torque complexity remained unchanged in lengthened condition. This could be due to compensatory mechanisms such as the elastic properties of actin-myosin cross bridges^51^, the passive tension generated by the connective tissue components of skeletal muscle^48,52,53^, and the higher calcium sensitivity at longer sarcomere lengths^51^, that allowed the neuromuscular system to preserve its ability to adapt the motor output and maintain the level of force production required by task demands, resulting in similar torque regularity values (i.e. no changes in SampEn).

Moreover, evidence suggests that the degree of knee flexion used in our study (~ 99°, OA + 30º) does not substantially affect torque production, as quadriceps sarcomere lengths at these angles remain within the plateau region of the length–tension curve, while force production impairments due to sarcomere elongation are typically observed only at more extreme joint angles^54^. This supports the idea that the neuromuscular system’s adaptability was preserved under our lengthened condition. However, we hypothesise that further increases in muscle lengthening could eventually lead to greater torque regularity, aligning with the initially proposed inverted U-shape relationship between torque complexity and joint angle.

Furthermore, the literature suggests that complexity- (SampEn) and magnitude-based measures (CV) provide distinct information: while magnitude-based measures represent the torque steadiness, i.e. the ability to accurately produce muscular torque, complexity-based measures represent the adaptability of the neuromuscular system, i.e. the ability to adapt motor output to the task demands^5^. Our findings support this distinction since we observed that, contrary to SampEn, CV increased in both lengthened (OA + 30º) and shortened (OA-30º) positions, suggesting that deviations from the OA impair the neuromuscular system’s ability to maintain torque steadiness and, consequently, produce precise movement. Notably, this result aligns with previous work showing an increased CV at shortened position^23^. However, we found that in lengthened position (OA + 30º), the CV was also increased which could be related methodological differences between experiments. In the present study, we explored a broader angle range (up to ~ 99º), beyond the 90º used in the previous study. These findings suggest that torque steadiness could be preserved within a mid-range of knee joint angles but declines when the joint is placed in extreme flexion or extension positions (OA-30º and OA + 30º).

Importantly, in the present study, we extended the work of Sosnoff and colleagues^23^ by assessing the agonist-antagonist co-contraction index (CCi), a measure of intermuscular coordination, through surface EMG. Overall, we found the lowest CCi values at OA and the highest values in either shortened or lengthened positions. Our results are consistent with Wu and colleagues’ findings^35^, although their limited angle range (60º and 90º) prevent us to take any further conclusion regarding other testing angles. We propose that increased knee flexion enhances antagonist activation, increasing CCi. Conversely, in the shortened positions, increased CCi may reflect a neuromuscular strategy to stabilise the knee joint due to the role of knee flexors as dynamic stabilizers of the tibia, preventing excessive anterior translation during knee extension^55^. Further, this protective strategy may have constrained joint behaviour, contributing to more regular output, as reflected by lower SampEn. Interestingly, CCi followed a similar pattern to CV across joint angles. This suggests that greater co-contraction at extreme joint positions may impair force regulation by reducing the individuals’ capacity to produce steady and complex outputs.

Although CCi offers valuable insight, it reflects only one aspect of intermuscular coordination. Future studies could incorporate other measures such as EMG-EMG coherence analysis, a frequency-domain approach to assess common synaptic input across muscles^56–58^. which could clarify the role of synergistic coordination in motor control across different joint angles. Likewise, techniques such as Transcranial Magnetic Stimulation, Peripheral Nerve Stimulation or High-density Electromyography could elucidate how corticospinal drive and motor unit behaviour contribute to force regulation across joint angles^23,59–61^. Furthermore, including only male participants is a limitation of the present study. Considering the emerging evidence of sex-based differences in force control^62–64^, future studies should explore whether joint angle affects torque complexity differently based on an individual’s sex.

The present study extended previous research that investigated the influence of joint angle on toque regulation by demonstrating a decrease in torque complexity with the shortening of the knee extensors. Moreover, by showing that torque complexity varies with joint angle, and, importantly, differs from changes in magnitude of variability we highlight the unique contribution of complexity-based metrics to understanding motor control. These findings suggest that torque complexity may serve as a sensitive biomarker for neuromuscular adaptability, with potential applications in rehabilitation and sports performance optimization, namely, by informing targeted interventions to preserve or enhance motor adaptability in clinical and athletic populations.

## Data Availability

The datasets generated during and/or analysed during the current study are available from the corresponding author on reasonable request.

## Code availability

All Matlab^®^ R2023a codes are deposited in *Zenodo* repository [^65^] and available on reasonable request.
